# Serine Biosynthesis Pathway Supports MYC–miR-494–EZH2 Feed-Forward Circuit Necessary to Maintain Metabolic and Epigenetic Reprogramming of Burkitt Lymphoma Cells

**DOI:** 10.3390/cancers12030580

**Published:** 2020-03-03

**Authors:** Emilia Białopiotrowicz, Monika Noyszewska-Kania, Neli Kachamakova-Trojanowska, Agnieszka Łoboda, Magdalena Cybulska, Aleksandra Grochowska, Michał Kopczyński, Michał Mikula, Monika Prochorec-Sobieszek, Małgorzata Firczuk, Agnieszka Graczyk-Jarzynka, Radosław Zagożdżon, Adam Ząbek, Piotr Młynarz, Józef Dulak, Patryk Górniak, Maciej Szydłowski, Karolina Pyziak, Justyna Martyka, Agnieszka Sroka-Porada, Ewa Jabłońska, Anna Polak, Piotr Kowalczyk, Anna Szumera-Ciećkiewicz, Bjoern Chapuy, Tomasz Rzymski, Krzysztof Brzózka, Przemysław Juszczyński

**Affiliations:** 1 Department of Experimental Hematology, Institute of Hematology and Transfusion Medicine, 02-776 Warsaw, Poland; ebialopiotrowicz@ihit.waw.pl (E.B.); noyszewska@gmail.com (M.N.-K.); pgorniak@ihit.waw.pl (P.G.); mszydlowski@ihit.waw.pl (M.S.); ewa_jab@wp.pl (E.J.); apolak@ihit.waw.pl (A.P.); 2 Department of Medical Biotechnology, Faculty of Biochemistry, Biophysics and Biotechnology, Jagiellonian University, 30-387 Cracow, Poland; neli.kachamakova-trojanowska@uj.edu.pl (N.K.-T.); agnieszka.loboda@uj.edu.pl (A.Ł.); jozef.dulak@uj.edu.pl (J.D.); 3 Malopolska Centre of Biotechnology, Jagiellonian University, 30-387 Cracow, Poland; 4 Department of Genetics, Maria Sklodowska-Curie National Research Institute of Oncology, 02-781 Warsaw, Poland; mcybulska@coi.pl (M.C.); grochowska.am@gmail.com (A.G.); kopczynski.mi@gmail.com (M.K.); michal.mikula@coi.pl (M.M.); 5 Department of Diagnostic Hematology, Institute of Hematology and Transfusion Medicine, 02-776 Warsaw, Poland; monika.prochorec@interia.pl (M.P.-S.); szumann@gmail.com (A.S.-C.); 6 Department of Immunology, Medical University of Warsaw, 02-097 Warsaw, Poland; mfirczuk@wum.edu.pl (M.F.); agnieszka.graczyk-jarzynka@wum.edu.pl (A.G.-J.); 7 Department of Clinical Immunology, Medical University of Warsaw, 02-006 Warsaw, Poland; radoslaw.zagozdzon@wum.edu.pl; 8 Department of Bioorganic Chemistry, Wroclaw University of Technology, 50-370 Wroclaw, Poland; adam.zabek@pwr.edu.pl (A.Z.); piotr.mlynarz@pwr.edu.pl (P.M.); 9 Ryvu Therapeutics S.A., 30-348 Cracow, Poland; karolina.pyziak@selvita.com (K.P.); justyna.martyka@selvita.com (J.M.); agnieszka.sroka-porada@ryvu.com (A.S.-P.); kowalczyk_piotr@wp.pl (P.K.); tomasz.rzymski@selvita.com (T.R.); krzysztof.brzozka@selvita.com (K.B.); 10 Department of Hematology and Oncology, University Medical Center Göttingen, 37-075 Göttingen, Germany; bjoern.chapuy@med.uni-goettingen.de

**Keywords:** Burkitt lymphoma, MYC, serine biosynthesis pathway, metabolism

## Abstract

Burkitt lymphoma (BL) is a rapidly growing tumor, characterized by high anabolic requirements. The *MYC* oncogene plays a central role in the pathogenesis of this malignancy, controlling genes involved in apoptosis, proliferation, and cellular metabolism. Serine biosynthesis pathway (SBP) couples glycolysis to folate and methionine cycles, supporting biosynthesis of certain amino acids, nucleotides, glutathione, and a methyl group donor, S-adenosylmethionine (SAM). We report that BLs overexpress SBP enzymes, phosphoglycerate dehydrogenase (PHGDH) and phosphoserine aminotransferase 1 (PSAT1). Both genes are controlled by the MYC-dependent ATF4 transcription factor. Genetic ablation of PHGDH/PSAT1 or chemical PHGDH inhibition with NCT-503 decreased BL cell lines proliferation and clonogenicity. NCT-503 reduced glutathione level, increased reactive oxygen species abundance, and induced apoptosis. Consistent with the role of SAM as a methyl donor, NCT-503 decreased DNA and histone methylation, and led to the re-expression of *ID4*, *KLF4*, *CDKN2B* and *TXNIP* tumor suppressors. High H3K27me3 level is known to repress the MYC negative regulator miR-494. NCT-503 decreased H3K27me3 abundance, increased the miR-494 level, and reduced the expression of MYC and MYC-dependent histone methyltransferase, EZH2. Surprisingly, chemical/genetic disruption of SBP did not delay BL and breast cancer xenografts growth, suggesting the existence of mechanisms compensating the PHGDH/PSAT1 absence in vivo.

## 1. Introduction

Burkitt lymphoma (BL) is an aggressive B-cell non-Hodgkin lymphoma (NHL) and one of the fastest-growing human tumors with nearly 100% growth fraction and the doubling time reaching only 25 hours [1]. BL is the most common NHL among children, accounting for 50% of all lymphoma cases. Its incidence decreases in older age groups, constituting 1–2% of NHL cases in adults [2,3,4]. It is estimated that 90% of children with BL are cured with intensive chemotherapy, albeit at the cost of considerable toxicity and treatment-related complications [5]. Despite relatively good prognosis for the majority of patients, treatment options for relapsed and refractory BL remain limited, and median overall survival in these cases usually does not exceed 3 months [5,6,7]. Thus, efficient and safe BL therapy is an urgent and unmet clinical need.

The genetic hallmark of BL cells is the presence of chromosomal translocations, t(8;14), t(8;22), or t(2;8), that juxtapose MYC to the heavy- or light-chain immunoglobulin gene regulatory regions, leading to MYC overexpression [8,9]. The *MYC* gene encodes a leucine zipper transcription factor that regulates the expression of a broad spectrum of genes involved in cell proliferation, metabolism, growth, angiogenesis, metastasis, genomic instability, and stem cell self-renewal and differentiation [10]. Since most MYC-driven cancers, including BL, remain addicted to this oncogene, MYC inhibition appears to be an attractive therapeutic strategy. However, attempts of targeting MYC directly have been largely unsuccessful due to the undruggable protein structure. Instead, molecular pathways involved in MYC post-transcriptional/post-translational regulation have been intensively screened as new approaches to treat BL and other MYC-driven tumors [10,11]. 

MYC impacts many facets of tumor cell metabolism, such as glycolysis, glutaminolysis, and lipid and nucleotide synthesis, and thus affords metabolic flexibility to cancer cells in unfavorable conditions [12]. In a recent, unbiased metabolomic–proteomic screen, BL cells have been shown to markedly upregulate one-carbon (1C) metabolism pathway [13]. One-carbon metabolism comprises of a series of reactions initiated by the folate cycle, which is essential for de novo nucleotide synthesis and regeneration of mitochondrial NADH, NADPH, and ATP [14]. The folate cycle is coupled to the methionine cycle, which generates methyl groups for cellular biosynthesis and methylation reactions and provides cysteine for the synthesis of glutathione. Hence, 1C metabolism is an important biosynthetic hub for dividing cells. 

Carbon flux necessary to fuel 1C metabolism is derived from serine, which is synthesized in three consecutive reactions. The first enzyme of the serine biosynthesis pathway (SBP), phosphoglycerate dehydrogenase (PHGDH), converts a glycolytic intermediate 3-phosphoglycerate into 3-phosphohydroxypyruvate, which is subsequently transformed into phosphoserine by phosphoserine aminotransferase 1 (PSAT1). The latter product is finally converted by phosphoserine phosphatase (PSPH) into serine. Overexpression of PHGDH and PSAT1 due to structural alterations or functional mechanisms was found in many types of cancers [15,16,17,18]. Importantly, some of these tumors are sensitive to targeted SBP inhibition [19,20]. Since BL is a metabolically demanding malignancy, we hypothesized that the serine biosynthesis pathway might play an important role in the tumor’s biology. Thus, we examined SBP enzymes expression and assessed the consequences of their inhibition in BL cells. 

We report herein that SBP enzymes PHGDH and PSAT1 are upregulated in BL via a MYC/ATF4-dependent mechanism. Genetic or chemical PHGDH/PSAT1 inhibition leads to decreased proliferation and clonogenicity of BL cells in vitro. Importantly, we characterize the mechanistic background of SBP inhibition toxicity and demonstrate that uncoupling SBP from downstream pathways leads to decreased DNA and histone methylation, decreased MYC abundance, and downregulation of EZH2 (enhancer of zeste homolog 2) histone methyltransferase. However, despite the promising consequences of PHGDH inhibition in vitro, xenografted tumors with genetically/chemically disrupted SBP grow similarly to the controls in vivo.

## 2. Results

### 2.1. Overexpression of PHGDH and PSAT1 in BL Cells is Driven by MYC and ATF4

**Figure 1:**
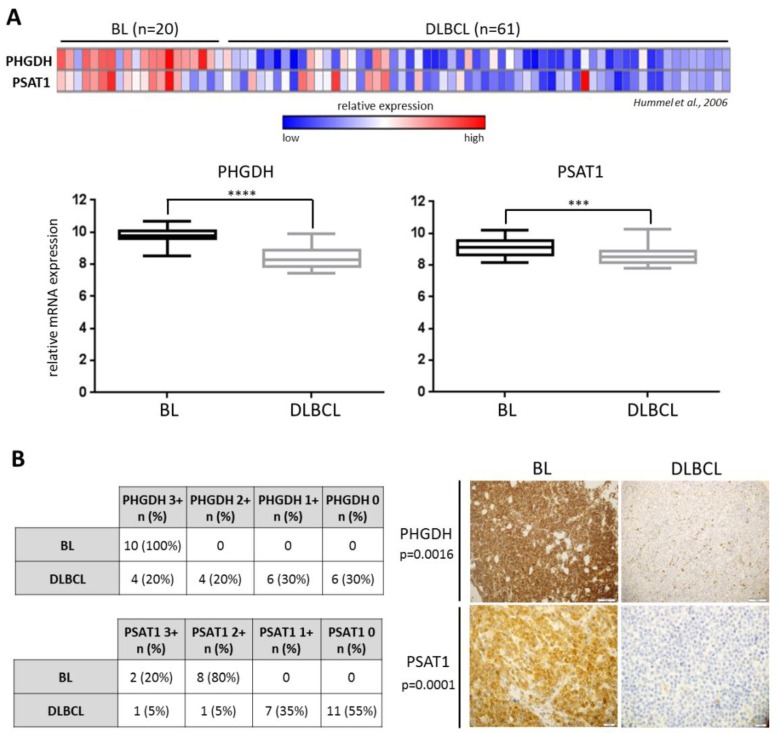
PHGDH and PSAT1 mRNA expression is significantly higher in Burkitt lymphoma (BL) than diffuse large B-cell lymphoma (DLBCL) primary cells. (**A**) Upper: Relative PHGDH and PSAT1 transcript abundance in primary tumor samples derived from 20 BL and 61 DLBCL patients. Each column represents a sample and each row refers to a gene probe; columns are ordered by tumor type (BL and DLBCL) as indicated. Color scale at the bottom indicates relative expression and standard deviations from the mean; *n*—number of patients. Lower: box plots illustrating differences in PHGDH and PSAT1 expression between BL and DLBCL primary samples; the median expression is indicated by the horizontal line, bars denote ± 25–75 percentile and whiskers indicate the range. Statistical analysis was performed using the Mann–Whitney test, **** for *p* < 0.0001 and *** for *p* < 0.001. (**B**) The expression of PHGDH and PSAT1 proteins is significantly higher in BL than DLBCL primary tumors. The immunohistochemical analysis included a group of 10 BL patients and 20 DLBCLs. The scale from 3+ to 0 refers to the staining intensity, where 3+ refers to strong staining and 0 means no staining. Cases 3+ or 2+ in IHC were considered high-expressers, whereas cases 1+ or 0 were considered low expressers. Numbers/frequencies of high- versus low-expressers in BL vs. DLBCL were compared using the two-sided Fisher exact test. Representative cases of PHGDH +/– and PSAT1 +/– BLs and DLBCLs diagnostic formalin-fixed paraffin-embedded slides are shown. Original magnification was × 40.

We first investigated the expression of SBP genes, *PHGDH* and *PSAT1*, in BL and another type of aggressive B-cell malignancy, diffuse large B-cell lymphoma (DLBCL), using a publicly available dataset [21]. The transcript abundance of both genes was significantly higher in BL than DLBCL samples (*p* < 0.0001 for *PHGDH* and *p* < 0.0003 for *PSAT1*, Figure 1A). Similar results were obtained when using the Oncomine microarray database and the dataset from Klapper et al. (Figure S1) [22]. We next assessed the expression of PSAT1 and PHGDH in a series of diagnostic BL and DLBCL biopsies using immunohistochemistry. BL tissue samples showed higher expression for PSAT1 and PHGDH when compared to DLBCLs (*p* = 0.0016 for PHGDH and *p* = 0.0001 for PSAT1, Figure 1B). 

**Figure 2:**
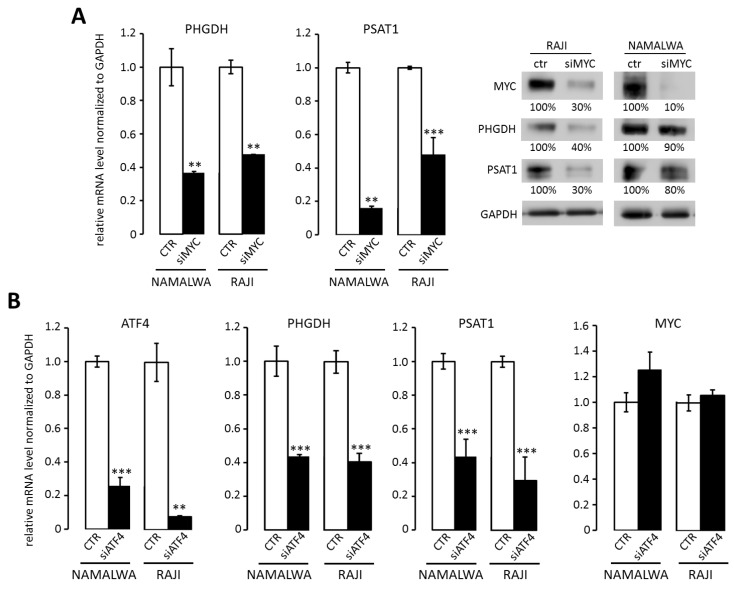
MYC regulates PHGDH and PSAT1 expression through ATF4 transcription factor. (**A**) MYC silencing decreases PHGDH and PSAT1 transcript and protein levels in RAJI and NAMALWA BL cell lines. Representative Western blots showing PHGDH and PSAT1 protein abundance after MYC silencing are shown. Protein abundance was quantified by densitometry using ImageJ software and presented as % of control. (**B**) The knockdown of ATF4 transcription factor decreases PHGDH and PSAT1 gene expression in BL cells. BL cells were transduced with siRNA targeting ATF4, incubated for 48 h, then the expression of ATF4, PHGDH, PSAT1, and MYC genes was analyzed with RQ-PCR. In (**A**) and (**B**), bar graphs represent averages of three independent experiments performed in technical triplicates, error bars represent standard deviation (SD). Statistical differences were assessed using *t*-test; * for *p* < 0.05; ** for *p* < 0.01 and *** for *p* < 0.001.

Since BL cells overexpress MYC, which is known to rewire tumor cell metabolism [12], we assessed the role of MYC in transcriptional regulation of SBP genes. MYC knockdown with siRNA led to the reduction in PHGDH and PSAT1 transcript and protein levels (Figure 2A). We found that the promoter regions of PSAT1 and PHGDH contain binding sites for MYC-controlled activating transcription factor 4 (ATF4), which was reported to regulate PSAT1 expression in breast cancer [23]. Accordingly, silencing of ATF4 decreased PHGDH and PSAT1 gene expression in RAJI and NAMALWA cells (Figure 2B).

To further evaluate the role of MYC in transcriptional regulation of SBP genes in primary tumors, we compared the transcript abundance of PHGDH and PSAT1 in two independent DLBCL cohorts annotated for MYC translocations/structural variants [21,24]. Expression of SBP genes exhibited moderate associations with MYC structural alterations in DLBCL (Figure S2A,B). In addition, the mRNA level for ATF4 was significantly lower in DLBCLs than BLs (Figure S2C). Taken together, these findings indicate that BLs overexpress SBP genes in a mechanism at least partially dependent on MYC and ATF4.

### 2.2. Knockdown of PSAT1 and PHGDH Impairs BL Cell Proliferation and Clonogenicity In Vitro

**Figure 3:**
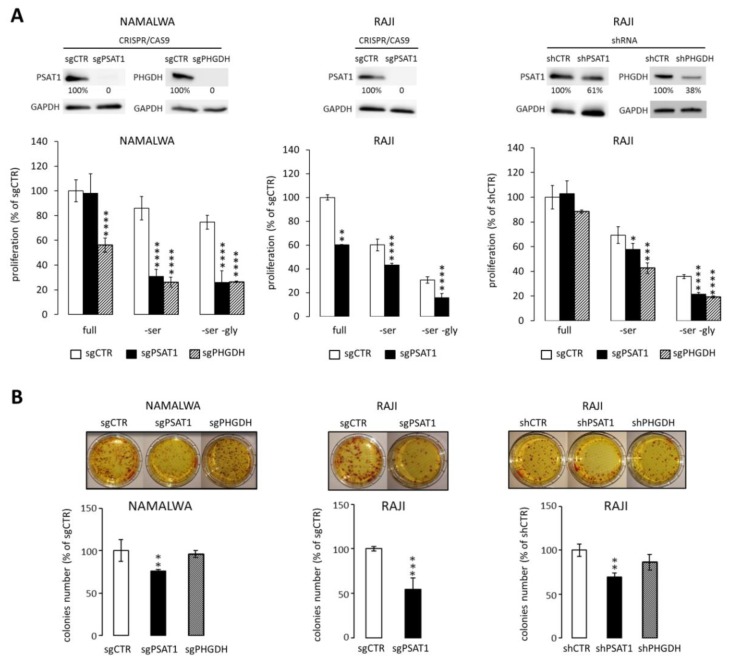
Disruption of PHGDH and PSAT1 expression decreases BL cell proliferation and clonogenicity. (**A**) Upper: CRISPR/Cas9 or shRNA-mediated targeting of PHGDH and PSAT1 in NAMALWA and RAJI cells. The protein expression of PSAT1/PHGDH was assessed relative to GAPDH using densitometry. Lower: proliferation of NAMALWA and RAJI cells with knocked-down expression of PHGDH or PSAT1. Cells were grown in either full, Ser- or Ser/Gly-depleted medium. Cell proliferation was assessed using MTS assay and presented relative to control cells (sgCTR or shCTR) grown in the full medium. (**B**) PHGDH and PSAT1 genetic inhibition decreases BL cells’ clonogenic potential. Bar graphs in (**A**) and (**B**) represent the averages from three independent experiments performed in triplicates ± SD. Statistical differences were assessed using *t*-test; * for *p* < 0.05; ** for *p* < 0.01 and *** for *p* < 0.001.

Overexpression of PHGDH and PSAT1 suggests that BL cells may be specifically reliant on serine metabolism for their growth and survival. To address this hypothesis, we generated NAMALWA and RAJI cell lines with PHGDH and PSAT1 knockout using the CRISPR/Cas9 method (Figure 3A). Of note, we did not obtain PHGDH knockout in RAJI, since the cells died rapidly during the selection procedure. These observations suggest that complete silencing of PHGDH might be cytotoxic to BL cells, at least in a subset of tumors. Thus, we used shRNA to decrease PSAT1 and PHGDH expression, expecting that residual PHGDH expression would suffice to maintain cell viability (Figure 3A). To evaluate the effect of PSAT1/PHGDH inhibition on cellular proliferation, the generated BL cell lines were grown in either full, serine-free (-Ser), or serine- and glycine-free medium (-Ser, -Gly). Exclusion of these amino acids from the culture medium partially decreased the proliferation of control RAJI and NAMALWA cells (Figure 3A). Complete inhibition of PSAT1 and PHGDH gene expression resulted in decreased cell proliferation, which decreased even further in Ser- and Ser/Gly-depleted media (Figure 3A). In cell lines with residual PSAT1/PHGDH expression (shPSAT1 RAJI, shPHGDH RAJI) no major effect on cell proliferation in full medium was observed. However, cell proliferation was significantly decreased in Ser- and Ser/Gly-depleted media (Figure 3A). Finally, PSAT1 knockdown significantly decreased the clonogenicity of NAMALWA and RAJI cell lines, whereas PHGDH disruption produced less pronounced effects (Figure 3B).

### 2.3. PHGDH Inhibitor NCT-503 Decreases Proliferation and Clonogenicity of BL Cells, Evokes Oxidative Stress, and Induces Apoptosis

**Figure 4:**
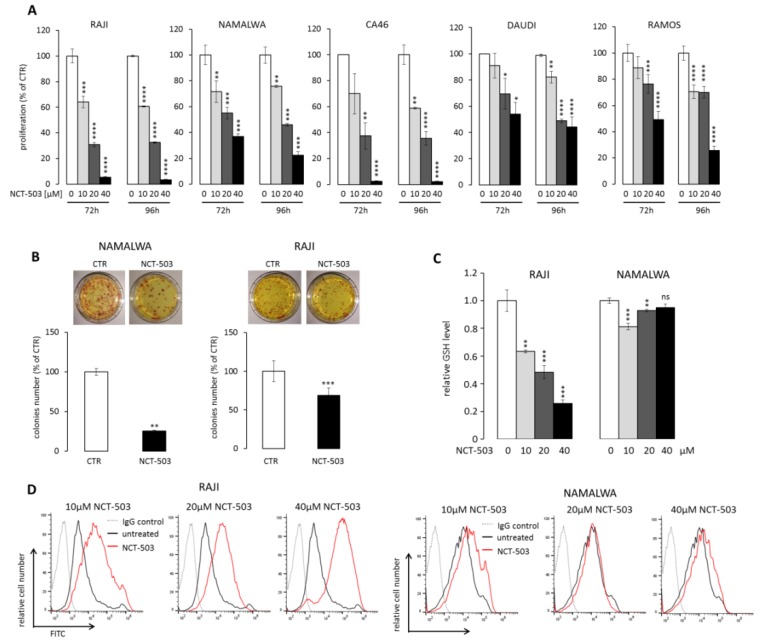
PHGDH inhibitor NCT-503 decreases viability and clonogenicity, generates oxidative stress, and induces apoptosis. (**A**) NCT-503 decreases proliferation of Burkitt cell lines. Cells were incubated with increasing doses of NCT-503 for 72–96 h; thereafter, cell viability was assessed using MTS assay. (**B**) PHGDH inhibition with NCT-503 impairs BL cell lines’ clonogenic potential. Cells were grown in the semi-solid medium in the presence or absence of NCT-503 (40 μM) as described (see Section 4). Example photographs of plates/colonies are shown. (**C**) NCT-503 decreases cellular glutathione level (GSH) in BL cells (10–40 µM, 24 h). (**D**) NCT-503 increases ROS content in RAJI and NAMALWA cells. Cells were incubated with NCT-503 for 24 hours. ROS content was measured by CM-H_2_DCFDA staining using flow cytometry. Representative histograms of three independent experiments are shown. In (**A**–**C**), graphs represent summary data from three independent experiments performed in triplicates. Statistical analysis was performed using the *t*-test; * for *p* < 0.05, ** for *p* < 0.01, *** for *p* < 0.001 and **** for *p* < 0.0001.

Given decreased viability and clonogenicity of BL cell lines with a targeted disruption of SBP enzymes, we investigated whether chemical SBP inhibition could represent a potential therapeutic approach in BL. As PSAT1 inhibitors are not commercially available, we targeted SBP using a PHGDH inhibitor, NCT-503. First, to confirm the specificity of NCT-503 inhibitor, we mutated the key PHGDH cysteine 234 to serine (C234S). This mutation had been previously shown to decrease NCT-503 binding to PHGDH and restore serine flux in breast cancer cells [18]. RAJI cells expressing C234S mutant were significantly less sensitive than control cells, confirming the on-target activity of the inhibitor (Figure S3 and Supplementary Methods). Next, we incubated five BL cell lines (RAJI, NAMALWA, CA46, DAUDI, and RAMOS) with increasing concentrations of NCT-503 in full medium for 72–96 h and then assessed cellular proliferation. PHGDH inhibitor significantly reduced cell growth of all analyzed cell lines in a dose-dependent manner (Figure 4A). Incubation of two sensitive BL cell lines, RAJI and NAMALWA, with 40 μM NCT-503 also significantly decreased their clonogenic potential, as shown in Figure 4B. 

Serine is a substrate for glycine production, which is utilized for glutathione (GSH) biosynthesis. As GSH is a key cellular reactive oxygen species (ROS) scavenger, we assessed the consequences of SBP inhibition for cellular GSH and ROS contents. RAJI cells, and to a lesser degree NAMALWA cells, treated with NCT-503 exhibited reduced GSH level and markedly elevated ROS activity (Figure 4C,D). Given the pro-oxidative activity of NCT-503, we asked whether prolonged incubation with the inhibitor would trigger cell death in BL cells. In four cell lines (RAJI, RAMOS, NAMALWA, and CA46) incubation with NCT-503 induced 24.7%–71.8% apoptosis, whereas in DAUDI cells, despite significant cytostatic effect, the inhibitor did not trigger cell death (Figure S4).

PSAT1-catalyzed reaction utilizes 3-phoshohydroxypyruvate and glutamate as substrates to produce α-ketoglutarate and phosphoserine [25]. Thus, inhibition of glutaminase (GLS) might deplete PSAT1 from a substrate (glutamine) and synergize with SBP inhibition. In line with this hypothesis, glutamine depletion increased the sensitivity of BL cells to NCT-503 (Figure S5A). Similarly, chemical glutaminase inhibitor CB-839 (currently in clinical trials for non-Hodgkin lymphomas) exhibited synergistic or additive effects with NCT-503 for selected dose combinations in four out of five cell lines (Figure S5B). 

### 2.4. NCT-503 Decreases Histone and DNA Methylation and Causes Re-expression of Tumor Suppressor Genes

**Figure 5:**
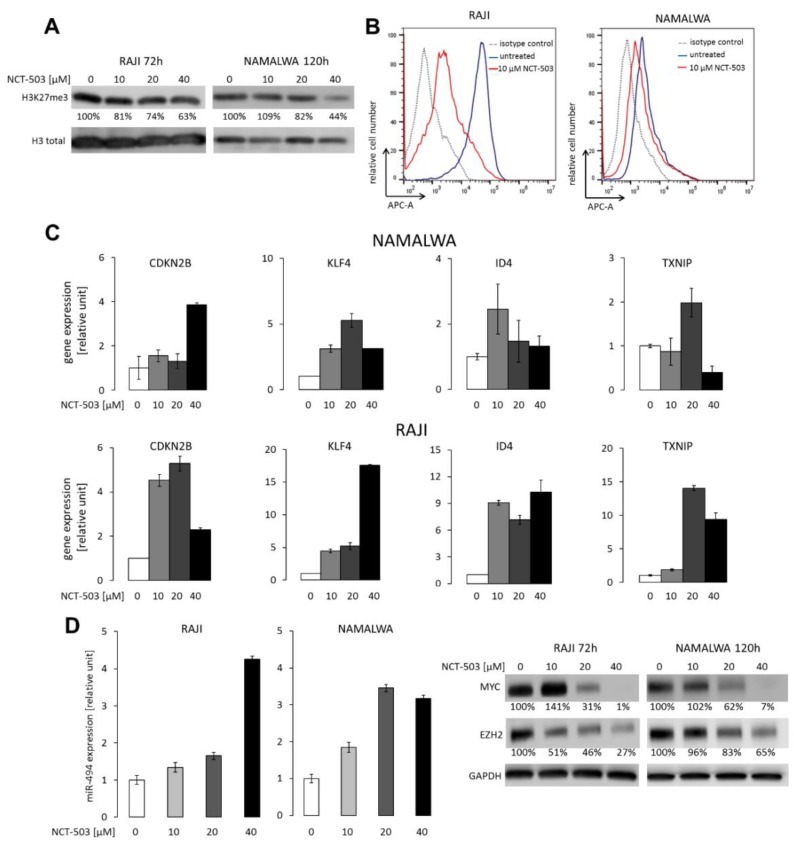
PHGDH inhibition with NCT-503 affects histone H3 and DNA methylation, causes re-expression of tumor suppressor genes, and decreases MYC and EZH2 levels. (**A**) Decreased H3K27me3 after 96 and 120 h incubation with NCT-503 in RAJI and NAMALWA, respectively. Representative Western blot of 3 independent experiments with densitometry (% of control) is shown. Histone H3 was used as a loading control. (**B**) NCT-503 decreases global DNA methylation. RAJI and NAMALWA cells were incubated with 10 µM NCT-503 for 72 h, then stained for 5-methylcytosine and analyzed with flow cytometry. Representative histograms of three independent experiments are shown. (**C**) Chemical inhibition of PHGDH restores the expression of tumor suppressor genes in RAJI and NAMALWA. Cells were incubated with different doses of NCT-503 (10–40 µM, 72 h). Thereafter, mRNA abundance of CDKN2B, KLF4, ID4, and TXNIP was assessed using RQ-PCR. (**D**) Chemical inhibition of PHGDH with NCT-503 induces miR-494 expression and leads to decreased EZH2 and MYC protein levels. The expression of miR-494 was assessed using RQ-PCR. In (**C**) and (**D**), bars indicate means ± SD from a representative of three independent experiments performed in triplicates.

Burkitt lymphoma is characterized by the deregulation of DNA methylation switching certain tumor suppressor genes off [26,27,28]. Serine biosynthesis pathway is linked to the methionine cycle, responsible for the production of S-adenosylmethionine (SAM), a universal methyl group donor in cellular DNA and histone methylation reactions. Thus, we hypothesized that SBP inhibition would impact SAM production, decrease DNA and histone methylation, and consequently perturb BL gene expression program. To address this hypothesis, we assessed the level of trimethylation of histone H3 lysine 27 (H3K27me3) in RAJI and NAMALWA after incubation with 10–40 µM NCT-503 and found a significant, dose-dependent decrease in the abundance of this mark in inhibitor-treated cells (Figure 5A). NCT-503 also markedly diminished the level of global DNA methylation, as measured by 5-methylcytosine staining (Figure 5B). Importantly, in parallel to changes in DNA and histone H3 methylation status, we found a marked re-expression of methylation-regulated tumor suppressor genes: *CDK2NB*, *KLF4*, *ID4*, and *TXNIP* (Figure 5C). 

Increased H3K27me3 is required to support MYC translation via repressing miR-494 expression [29]. Since SBP disruption with NCT-503 resulted in decreased H3K27me3 level, we further assessed miR-494 and MYC expression in BL cells incubated with the inhibitor. We found a significant, dose-dependent miR-494 upregulation, which was accompanied by the reductions in MYC protein levels in both RAJI and NAMALWA cell lines (Figure 5D). MYC is known to negatively regulate miR-26a, which targets the Enhancer of zeste homolog 2 (EZH2) methyltransferase responsible for maintaining H3K27 trimethylation [29,30]. Accordingly, the decrease in the MYC protein levels resulted in EZH2 downregulation, confirming a feed-forward regulation mechanism linking these two proteins in BL cells (Figure 5D) [29].

### 2.5. PHGDH and PSAT1 Inhibition Does Not Impair BL and Breast Cancer Growth In Vivo

**Figure 6:**
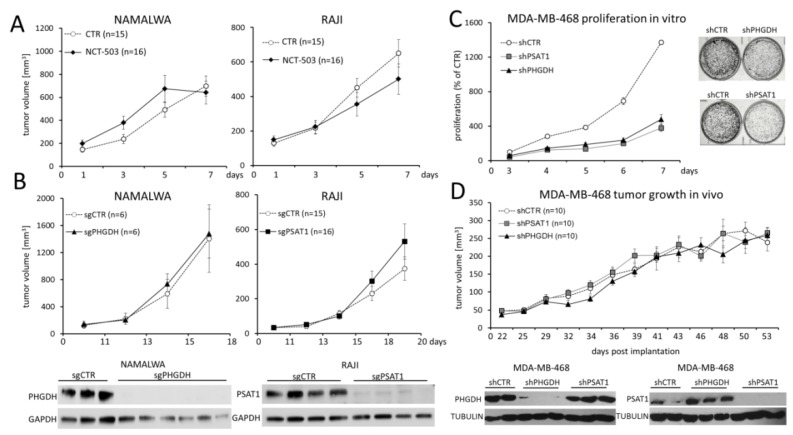
Inhibition of SBP in vivo does not affect the growth of NAMALWA and RAJI tumors. (**A**) Xenograft growth kinetics in mice inoculated with RAJI or NAMALWA cells and treated with NCT-503 or NCT-503 inactive control (CTR), each at a dose of 40 mg/kg/day intraperitoneally. (**B**) The effect of PHGDH (left) and PSAT1 (right) genetic knockout on the xenograft growth. (**C**) Genetic PHGDH and PSAT1 knockdown with shRNA impairs MDA-MB-468 breast cancer cell line proliferation and clonogenicity in serine-deficient medium in vitro (upper). The graph in (**C**) shows means ± SD from two independent experiments performed in triplicates. **** *p* < 0.0001 for shCTR vs. shPSAT1 and shCTR vs. shPHGDH for all analyzed days. Statistical analysis was performed using ANOVA. (**D**) PHGDH/PSAT1 knockdown does not impact the growth of MDA-MB-468 xenografts in vivo. Graphs in (**A**,**B**,**D**) represent means ± standard errors of the means; “*n*” refers to the number of mice in each experimental group. Decreased expression of PHGDH and PSAT1 in BL and MDA-MB-468 xenografts was confirmed by immunoblotting; representative cases from each experimental group are shown.

Since targeting SBP using PHGDH inhibitor NCT-503 exhibited encouraging results in in vitro studies, we next evaluated the therapeutic potential of chemical SBP disruption in vivo. For this purpose, mice were subcutaneously inoculated with NAMALWA or RAJI cells, and xenograft-bearing animals with tumor volumes exceeding 150 mm^3^ were daily injected intraperitoneally with 40 mg/kg NCT-503. Surprisingly, and in contrast to in vitro experiments, NCT-503 did not delay RAJI or NAMALWA tumor growth when compared to the control groups receiving NCT-503 inactive control, as shown in Figure 6A.

To exclude the contribution of suboptimal pharmacokinetics of NCT-503 to the lack of the therapeutic activity in the BL xenograft model, we next inoculated mice with NAMALWA cells with CRISPR/Cas9-mediated PHGDH knockout and compared the growth of the obtained tumors to those with unaffected PHGDH expression. Also in this experiment, the growth of NAMALWA tumors with blocked PHGDH was not delayed when compared to the controls (Figure 6B, left). Lack of PHGDH protein expression in the knockout tumors was confirmed with the Western blot (Figure 6B, left).

We next asked whether targeting PSAT1 would exhibit higher therapeutic potential. Analogously to the previously described experiment, mice were subcutaneously injected with RAJI cells with PSAT1 knockout or control cells. Similarly to PHGDH disruption, we found that PSAT1 knockout did not affect the tumor growth in vivo (Figure 6B, right). To gain a better insight into the metabolic consequences of PSAT1 disruption in vivo, the extracts from PSAT1 knockout and control xenografts were used to assess the content of 40 small molecule metabolites using nuclear magnetic resonance (NMR) spectroscopy. Although PSAT1-knockout and control tumors exhibited no difference in growth in vivo, they formed separate branches in this analysis, highlighting metabolic differences between these groups (Figure S6).

To further explore the therapeutic potential of SBP targeting in vivo, we employed a breast cancer xenograft model (MDA-MB-468 cell line) [31]. Genetic targeting of PSAT1 and PHGDH using shRNA caused a significant decrease in proliferation and clonogenicity of malignant cells in the serine-deprived medium as compared to the controls in vitro (Figure 6C). However, tumors with PSAT1 or PHGDH knockdown grew similarly to those with unaffected PSAT1/PHGDH expression in xenotransplant-bearing mice (Figure 6D). Importantly, in the explanted tumors with PSAT1 or PHGDH knockdown, there was no re-expression of the enzymes (Figure 6D), indicating that disruption of SBP in the breast cancer cell model is not sufficient to delay the growth of an established tumor.

## 3. Discussion

As rapidly growing tumors must support their anabolic needs, metabolic reprogramming is one of the cancer hallmarks. Cancer metabolic reprogramming involves three fundamental alterations: changes in bioenergetics and energy source utilization, enhanced biosynthesis, and redox balance, which together improve cellular fitness to provide a selective advantage for a developing tumor. These metabolic alterations also affect tumor immunogenicity and sensitivity to chemotherapy, placing altered metabolism in the spotlight as a putative therapeutic target. However, therapeutic exploitation of cancer’s metabolic addiction requires pinpointing mechanisms responsible for metabolic reprogramming and identification of enzymes that account for these changes.

Burkitt lymphoma is one of the most rapidly growing human tumors, suggesting that identification of pathways supporting its high metabolic needs would provide a viable therapeutic strategy. Metabolic reprogramming of BL cells is strongly regulated by the MYC oncogene. MYC has been recently shown to switch the neoplastic cells toward glutamine metabolism, opening the way to target glutaminolytic enzymes in MYC-driven tumors [32]. Moreover, MYC also regulates a monocarboxylic acid transporter 1 (MCT1), which facilitates the removal of metabolic end products, such as lactate. Inhibition of MCT1 was shown to impair MYC-driven lymphomagenesis [33]. Both glutaminase and MCT1 inhibitors are being tested in clinical trials [34,35]. 

A recent comparative study revealed that BL cells upregulate 1C metabolism [13]. In fact, targeting 1C-coupled folate cycle with antifolates (e.g., methotrexate) has been exploited in BL therapy for decades. Since 1C metabolism derives its carbons from serine, we evaluated herein the expression of serine biosynthetic pathway enzymes and assessed the biological consequences of their inhibition in BL cells. We first demonstrated that SBP enzymes are overexpressed in BL, compared to another aggressive lymphoma, DLBCL, and transcriptionally controlled by MYC via ATF4. A moderate correlation between MYC structural alterations and SBP genes expression was also observed in two independent series of DLBCLs, despite the lower level of ATF4 in DLBCLs than BLs. Unlike the case of serine hydroxymethyltransferase (SHMT), 5’ regulatory regions of PHGDH and PSAT1 do not directly bind MYC [36]. Thus, MYC appears to be an indirect regulator of SBP enzymes, acting, at least partially, through ATF4. Since ATF4 is regulated by MYC-dependent and MYC-independent mechanisms [37], these disease-specific differences in regulation of SBP genes can be likely explained by nonlinear relationships between ATF4 and MYC. 

Chemical or genetic inhibition of PHGDH and PSAT1 significantly affected BL cell lines’ proliferation and clonogenic potential in vitro and eventually induced apoptosis in the majority of cell lines. Mechanistically, inhibition of SBP decreased cellular GSH pool, induced oxidative stress, and decreased DNA and histone H3 methylation. As GSH synthesis and SAM generation are coupled to 1C metabolism, these observations suggest that inhibition of SBP decreased metabolic flux through folate and methionine pathways. 

**Figure 7:**
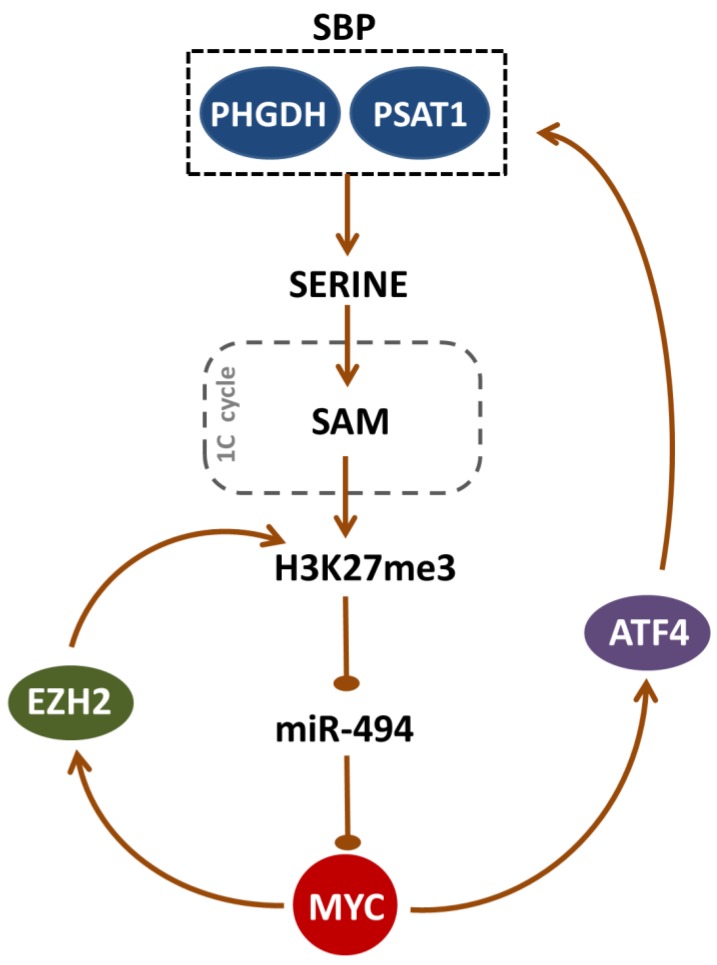
The reciprocal link between the serine biosynthesis pathway (SBP) and MYC oncogene. High PHGDH and PSAT1 activity leads to increased serine production, which fuels 1C metabolism, including folate and methionine cycles. One-carbon units from serine are utilized in the methionine cycle for the production of S-adenosylmethionine (SAM), which provides methyl groups for cellular methylation reactions, such as histone H3 trimethylation (H3K27me3). Increased H3K27me3 level is required to block miR-494 expression, which targets the transcription of MYC oncogene. MYC contributes to maintain high H3K27me3 level by indirectly regulating the expression of EZH2 methyltransferase and promoting the expression of SBP enzymes PHGHD and PSAT1 through ATF4 transcription factor. Sharp arrows: activation; blunt arrows: inhibition.

Importantly, the EZH2-mediated H3K27 trimethylation is required to maintain the MYC-targeting miR-494 in a repressed state [29]. This microRNA, along with EZH2 and MYC-regulated miR-26a targeting of EZH2, are the components of a feed-forward vicious loop maintaining high levels of MYC expression [29,30]. We showed that the inhibition of H3K27me3 by SBP pathway disruption in vitro led to a massive upregulation of miR-494 and, subsequently, downregulation of MYC. As EZH2 is regulated indirectly by MYC, decreased MYC expression reduced EZH2 abundance, perpetuating/amplifying the changes initiated by SBP blockade and finally leading to the disruption of the feed-forward mechanism. Thus, our work uncovers a mechanistic link between SBP enzymes and MYC, involving EZH2 and miR-494 (Figure 7). As MYC is the pivotal pathogenetic driver of BL, but a hitherto undruggable target, these observations highlight a potential approach to indirect MYC targeting.

In addition to serine production, SBP might provide additional benefits to a cancer cell. For example, the byproduct of PSAT1-catalyzed reaction is α-ketoglutarate, the anaplerotic substrate of the tricarboxylic acid cycle (TCA). The knockdown of SBP enzymes was shown to cause a considerable drop in α-ketoglutarate level in the breast cancer model, while intracellular serine level remained unaffected [31]. Moreover, PHGDH is capable of producing 2-hydroxyglutarate (2-HG), an oncometabolite inhibiting DNA demethylases and leading to DNA hypermethylation [38]. Thus, apart from affecting intracellular serine level, SBP might also have serine-independent functions in tumorigenesis. 

Despite these vital functions of SBP in BL biology, surprisingly, our in vivo experiments using genetic or chemical disruption of PHGDH and PSAT1 did not demonstrate significant inhibition of BL growth. Although in vivo studies are key experiments in target validation and are considered a gold standard for this purpose, the executed in vivo experiments had certain limitations that must be taken into account in result interpretation.

First, CRISPR/Cas9-generated cells with PHGDH/PSAT1 knockout are selected for adaptation to enzyme loss. During the selection process, cells incapable of adaptation or suffering from toxicity are eliminated and/or overgrown. Thus, obtained viable cell populations are a suboptimal model to demonstrate the toxicity of SBP disruption. 

Second, as serine is one of the most abundant amino acids in cell culture medium and blood [39], cells with disrupted endogenous SBP might rely on increased substrate import from the microenvironment. However, at least in vitro, extracellular serine did not mitigate the consequences of SBP alteration in BL. We found that the complete loss of PHGDH/PSAT1 expression significantly impaired the proliferation of BL cells in the full medium (containing serine), suggesting that extracellular availability of this amino acid cannot entirely compensate the biological consequences of PSAT1/PHGDH knockout. Alternatively, BL cells deprived of serine might engage additional cell-intrinsic defense/adaptation mechanisms, such as autophagy [40,41]. In line with this hypothesis, genetic PHGDH knockdown promoted mTOR-independent autophagy in embryonal carcinoma stem-like cells [42]. In the same cellular model, an mTOR inhibitor, rapamycin, further augmented autophagy and induced apoptosis [42]. These data suggest that combining SBP blockade with other molecular pathway inhibitor(s) could be more effective than targeting SBP alone. Importantly, explanted control and PSAT1-deleted tumors exhibit distinct metabolomic profiles. These differences indicate either that the PSAT1 loss cannot be effectively compensated or that knockout cells engage additional metabolic pathways that result in metabolomics dissimilarities but ensure cell survival.

Third, similarly to our report, in estrogen receptor (ER)-negative breast cancer cell lines and xenografts, distinct effects of PHGDH targeting in vitro and in vivo were observed previously [43]. Despite in vitro cytotoxicity of PHGDH inhibition, the enzyme silencing using inducible shRNA system in fully established breast cancer xenografts did not delay tumor growth when compared to the xenografts with unaltered PHGDH expression [43]. However, when PHGDH was inducibly turned off in small xenografts, tumor development was arrested [31]. These results suggest that PHGDH might be essential only at the early steps of breast cancer development [43]. 

Taken together, our studies extend the spectrum of known MYC-induced metabolomic programs and further underscore the role of MYC as the master regulator of metabolic reprogramming in Burkitt lymphoma. Furthermore, we identify a mutual link between MYC, EZH2, and serine biosynthesis pathway, sustaining high expression levels of each component of this feed-forward loop in BL cells. Importantly, targeting SBP in BL cells disrupts the feed-forward loop and decreases MYC and EZH2 abundance. Given the limitations of xenograft studies presented herein, further and more detailed in vivo studies, particularly combining SBP targeting with inhibitors of potential compensatory pathways, are warranted.

## 4. Materials and Methods 

### 4.1. Cell Lines, Cell Culture and Chemicals

All human Burkitt lymphoma cell lines were purchased from Deutsche Sammlung von Mikroorganismen und Zellkulturen (DSMZ). MDA-MB-468 breast cancer cell line was purchased from American Type Culture Collection (ATCC). BL cell lines were grown in RPMI-1640 medium supplemented with heat-inactivated 10% or 20% (RAMOS) fetal bovine serum (FBS), 100 U/mL penicillin, 100 U/mL streptomycin, and 25 mmol/L HEPES buffer (all from Lonza) at a density of 0.5–2.0 × 10^6^ cells/mL. MDA-MB-468 cell line was cultured in DMEM low glucose medium (Lonza) supplemented with FBS, penicillin, and streptomycin as above. All cells were grown in a humidified atmosphere at 37 °C with 5% CO_2_. 

PHGDH inhibitor NCT-503 (N-(4,6-dimethylpyridin-2-yl)-4-(4-(trifluoromethyl)benzyl) piperazine-1-carbothioamide) and NCT-503 inactive control (N-(4,6-dimethylpyridin-2-yl)-4-(pyridin-4-yl)piperazine-1-carbothioamide), unable to inhibit PHGDH) were purchased from Sigma-Aldrich. Glutaminase inhibitor CB-839 was purchased from MedChemExpress. All compounds were diluted in sterile dimethyl sulfoxide (DMSO) to obtain 10 mM stocks, aliquoted, and stored in 2–8 °C according to manufacturer’s recommendations.

### 4.2. CRISPR/Cas9 and RNAi Gene Targeting

To perform PSAT1/PHGDH knockout with CRISPR/Cas9 method, specific sgRNAs were designed using the GeCKO v.2 library and synthesized by Sigma Aldrich. The sgRNAs sequences are given in Table S1. The pairs of sgRNA were annealed, phosphorylated on 5’ ends, and cloned into lentiCRISPR v.2 vector (a gift from Feng Zhang, Addgene plasmid # 52961), previously digested with BsmBI restrictase (Fermentas). Generated plasmids along with psPAX2 and pMD2.G vectors (gifts from Didier Trono, Addgene #12260 and #12259) were introduced into HEK293T cells to produce lentivirus particles. After 24 h, the lentivirus-containing supernatant from HEK293T cells was filtered, centrifuged (3000× *g*, 16 h, 4 °C), mixed with 8 µg/mL polybrene (Sigma Aldrich), and added to BL cells for 24 h. Thereafter, the supernatant was replaced with fresh medium and the cells were grown for an additional 48 h. After that time, the cells underwent puromycin selection (2 µg/mL) and subcloning by limiting dilution. Individual subclones were assessed for PSAT1/PHGDH expression by immunoblotting. 

Short hairpin RNA (shRNA) sequences were designed using GeneScript siRNA Target Finder (https://www.genscript.com/tools/sirna-target-finder) and shRNA Sequence Designer (Clonetech). The sequences are given in Table S2. Designed oligos were synthesized (Sigma-Aldrich), annealed, digested with BamHI and EcoRI, phosphorylated with T4 polynucleotide kinase and cloned into pSIREN-RetroQ as described previously [44]. Obtained vectors were introduced into RAJI using retroviral infection followed by puromycin selection (2 µg/mL). 

MDA-MB-468 cells with PSAT1/PHGDH knockdown were generated using 29-mer shRNA constructs in pGFP-C-shLenti vectors (purchased from OriGene, Rockville, MD, USA), and pMD2.G and psPAX2as as described previously [45]. After 72 h from transduction, the GFP positive cells were sorted using MoFlo XDP cell sorter (Beckman-Coulter). 

MYC and ATF4 in BL cells were targeted using SMARTpool Accell siRNA from Dharmacon, according to the manufacturer’s protocol.

### 4.3. Real-Time Quantitative PCR (RQ-PCR)

RNA and microRNA were isolated using Gene MATRIX Universal RNA/miRNA Purification Kit (EURx, Gdansk, Poland) and transcribed to cDNA with Transcriptor Universal cDNA Master (Roche) and TaqMan MicroRNA Reverse Transcription (Applied Biosystems), respectively. Primer sequences are given in Table S3. Transcript abundance was measured using Itaq Universal SYBR Green Supermix (Bio-Rad) and LightCycler 480. GAPDH and U6 snRNA were used as housekeeping control genes for mRNA and miR-494, respectively. Relative transcript abundance was assessed using the 2^−ΔΔCT^ method as previously described [46]. 

### 4.4. Immunoblotting

Protein extracts were prepared using RIPA buffer supplemented with Protease Inhibitor and PhosSTOP Phosphatase Inhibitor Cocktail Tablets (Roche). Histones were isolated using Histone Extraction Kit (Abcam) according to the manufacturer’s instructions. Protein extracts were PAGE-separated, electrotransferred to PVDF membranes (Millipore), blocked in 5% bovine serum or nonfat milk, and then immunoblotted with primary and appropriate secondary antibodies (Table S4). Signals were detected and quantified as described elsewhere [47].

### 4.5. Patient Samples and Immunohistochemistry

A retrospective group of 10 BL and 20 diffuse large B-cell lymphoma, not otherwise specified (DLBCL, NOS) patients diagnosed according to 2016 WHO classification was enrolled for the study [48]. Lymph node biopsies were fixed in 10% formalin, routinely processed, and stained with hematoxylin and eosin. Immunohistochemistry was performed using automated immunohistochemical stainer (Dako Denmark A/S) and polyclonal antibodies: anti-PHGDH antibody (Sigma-Aldrich, dilution 1:750) and anti-PSAT1 (PA5-22124) antibody (ThermoFisher, dilution 1:500) (Table S4). EnVision Detection System (Dako Denmark A/S) was used for signal detection. Positive staining control for PHGDH and PSAT1 included the human kidney. Negative (isotype) controls were performed using ready to use FLEX Negative Control Mouse (code nr IR750; Dako Denmark A/S). A semiquantitative method for evaluation of immunostainings was applied including scoring system based on the combination of intensity (0—no staining; 1—weak; 2—intermediate; 3—strong staining) and percentage of cells, as described before [46]. Stained lymphoma sections were independently reviewed for PHGDH/PSAT1 expression by two hematopathologists. Samples were considered positive for PHGDH/PSAT1 when more than 30% of lymphoma cells expressed weak/intermediate/strong staining. All microphotographs were taken by a microscope DP72 Olympus BX63 camera (Olympus, Japan).

### 4.6. Cell Viability, Clonogenicity, Apoptosis, and ROS Quantification

BL cell viability/proliferation was assessed using CellTiter 96 AQueous Non-Radioactive Cell Proliferation assay (Promega) after growth in full RPMI-1640, or RPMI-1640 deprived of serine, or serine and glycine (ThermoFisher Scientific). The proliferation of MDA-MB-468 cell line was analyzed using MTT (3-(4,5-dimethylthiazol-2-yl)-2,5-diphenyltetrazolium bromide) assay (Sigma-Aldrich) after 3- to 7-day growth in MEM medium (HyClone) supplemented with 10% dialyzed FBS (HyClone) and deprived of serine.

For the clonogenic assay, BL cells were plated in triplicates at 1000 cells/dish in MethoCult H4035 (StemCell Technologies) with the addition of 8% RPMI-1640. After 21 days, colonies were stained with 3-(4,5-dimethylthiazol-2-yl)-5-(3-carboxymethoxyphenyl)-2-(4-sulfophenyl)-2H-tetrazolium and the total number of colonies was determined using Image J software (https://imagej.nih.gov/ij/). To assess the clonogenicity of MDA-MB-468, 1000 cells were seeded into each well of a six-well plate. On the next day, the medium was changed to the medium without serine and glycine, and cells were further grown for 8 days. Thereafter, the medium was removed, the cells were washed with PBS, fixed on ice with cold (−20 °C) 100% methanol for 20 minutes, and stained with 0.05% crystal violet (BioShop) in 20% methanol (20 min, room temperature). Pictures of the plates were taken using a Fusion FX5 XT camera (Vilber).

Apoptosis was assessed using Annexin V-PE/7AAD Apoptosis Detection Kit (BD Biosciences). The cells were analyzed with the Cytoflex flow cytometer (Beckman Coulter) and Flow-Jo software. Annexin V-positive and double Annexin V/7AAD-positive cells were considered apoptotic. Glutathione (GSH) level and reactive oxygen species (ROS) abundance were estimated using GSH/GSSG-Glo luminescent assay (Promega) and CM-H_2_DCFDA assay (Invitrogen), respectively. 

### 4.7. DNA Methylation

Cells were washed with PBS, fixed with CytoFix Buffer (BD Biosciences) for 15 min at room temperature (RT), centrifuged (600× *g*, 8 min) and permeabilized (0.5% Triton X-100 in PBS, 10 min). After washing, samples were incubated with 2 M hydrochloric acid (RT, 20min), neutralized with 100 mM Tris-HCl pH 8.0 (15 min, RT), centrifuged, washed with PBS, and incubated with anti-5-methylcytosine antibody or IgG isotype control (30 min, RT), (Table S4). After washing, cells were stained with APC-conjugated secondary antibody (30 min, in the dark), (Table S4), washed, and assessed using flow cytometry. Collected data were analyzed using Flow Jo software.

### 4.8. Tumor Xenografts

NOD/SCID mice (strains NSG/J and sgNOD.CB-17-Prkdc scid/Rj) were purchased from The Jackson Laboratory and Janvier Labs, respectively. Experiments were carried out in the animal facilities of Maria Skłodowska-Curie Institute—Oncology Centre, and the Jagiellonian University in accordance with the protocols approved by the Second Local Ethics Committee for Animal Experimentation in Warsaw (decision no. WAW2/032/2018) and the Second Local Institutional Animal Care and Use Committee (IACUC) in Cracow (approval number: 162/2017). To test the effect of NCT-503 on BL tumor growth, mice were inoculated subcutaneously with 1 × 10^7^ NAMALWA/RAJI cells. After the xenografts reached 150 mm^3^, the animals were randomized to receive NCT-503 or inactive control (each at a dose of 40 mg/kg/day) via intraperitoneal injections as described [18]. The tumor volume was measured for 7 consecutive days. For establishing PHGDH/PSAT1-knockout BL xenografts, mice were inoculated with 3 × 10^6^ PHGDH/PSAT1-depleted cells or control cell lines, prepared as described above. Tumor volume was measured from the 11th day after implantation. To investigate the effect of PHGDH/PSAT1 knockdown on the growth of breast cancer xenografts, mice were subcutaneously injected with 5 × 10^6^ PSAT1/PHGDH-knockdown or control MDA-MB-468 cells. Tumor growth was measured every 2–3 days for 50 consecutive days. Metabolomic analyses of explanted tumors are described in Appendix A.

### 4.9. Bioinformatic and Statistical Analysis

Gene expression datasets available in the public domain were used to assess PHGDH and PSAT1 abundance in lymphoma cells [21,22,24]. Data were analyzed and visualized using MORPHEUS software https://software.broadinstitute.org/morpheus/) and Oncomine website (https://www.oncomine.org/resource/login.html). Comparisons between variables and Pearson correlation between MYC and SBP transcripts abundance were performed with GraphPad Prism 6 software (GraphPad, La Jolla, CA, USA). For standard comparisons, the *t*-test was used, unless otherwise indicated; *p* < 0.05 was considered statistically significant.

## 5. Conclusions

Burkitt lymphomas, highly proliferative and metabolically demanding tumors, overexpress serine biosynthesis pathway (SBP) enzymes, phosphoglycerate dehydrogenase (PHGDH), and phosphoserine aminotransferase 1 (PSAT1). Expression of these proteins is driven by MYC oncogene, extending the spectrum of known MYC-induced metabolomic programs and further underscoring the role of MYC as the master regulator of metabolic reprogramming in BL. Inhibition of SBP decreased H3K27 trimethylation, prompting the induction of MYC-targeting miR-494, thus attenuating MYC abundance, and leading eventually to decreased expression of MYC-regulated EZH2. Therefore, SBP inhibition initiates metabolic–epigenetic alterations which break the self-reinforcing SBP–MYC–EZH2 loop, essential to maintain BL cell reprogramming. In line with these observations, genetic or chemical PHGDH/PSAT1 disruption in vitro promotes metabolic and epigenetic alterations, which change BL transcriptional program, generate oxidative stress, and induce apoptosis.

## Supplementary Materials

The following are available online at https://www.mdpi.com/2072-6694/12/3/580/s1, Figure S1: Comparison of PHGDH and PSAT1 transcript abundance in different B cell lymphomas, Figure S2: Expression of PSAT1 and PHGDH in MYC-rearranged and wild-type DLBCLs. Tumors; Correlation between MYC and PSAT1 or PHGDH transcript abundance in DLBCLs; Differences in ATF4 transcript levels between BL and DLBCL, Figure S3: RAJI control cells expressing PHGDH (shCTR) or RAJI cells with PHGDH knockdown (shPHGDH) were retrovirally transfected with pMIG:empty, pMIG:PHGDHwt or pMIG:PHGDH-C234S, sorted for GFP-positive cells and incubated with NCT-503 or NCT-503 inactive control for 72 h, Figure S4: NCT-503 induced apoptosis in BL cell lines, Figure S5: Interaction of glutamine depletion with SBP inhibition in BL cells, Figure S6: PSAT1 knockout changes the metabolomics of BL xenografts, Figure S7: Uncropped western blot images with molecular weight markers corresponding to Figure 2, Figure S8: Uncropped western blot images with molecular weight markers corresponding to Figure 3, Figure S9: Uncropped western blot images with molecular weight markers corresponding to Figure 5A,B, Figure S10: Uncropped western blot images with molecular weight markers corresponding to Figure 6B,D, Table S1: PHGDH and PSAT1 targeting sgRNA sequences, Table S2: PHGDH and PSAT1 targeting shRNA sequences, Table S3: Primers used for RQ-PCR, Table S4: Antibodies used in the study.

## Appendix A

Metabolomics analysis by nuclear magnetic resonance (NMR) spectroscopy. Metabolites from xenograft sections were extracted using LC/MS grade methanol (Merck) in TissueLyser LT (Qiagen), (10 min, 50 Hz) with stainless steel beads (5 mm). Then LC/MS grade water (Merck) was added and the samples were homogenized (50 Hz, 10 min), incubated at −80 °C for 2 h, thawed and homogenized again. Finally, the samples were centrifuged (10 min, 12 000 rpm, 4 °C) to collect the supernatant. Before NMR analysis the solvent was evaporated on SpeedVac (40 °C, 1500 rpm). NMR spectra were recorded at 300 K using an Avance III spectrometer (Bruker, Germany) operating at 700.58 MHz frequency. The spectra were processed with a 0.3 Hz line broadening, the phase was set manually and the baseline corrected using the MestReNova software (Mestrelab Research v 11.0). Each spectrum was corrected to the TSP signal = 0.0 ppm. Alignment of resonance signals was performed using an optimized algorithm (cow) and the icoshift algorithm implemented in the Matlab environment (v R2014a, Mathworks Inc.). All spectra were normalized to the TSP resonant signal and the mass of individual tissues.

Introduction of PHGDH with C234S mutation into shPHGDH RAJI cells. The sequences encoding wild type and mutated (C234S) PHGDH protein were ordered from ATG:biosynthetics basing on the information found in Addgene (#83902 and #83901). The DNA fragments were cloned into pMIG-GFP using XhoI and EcoRI restrictases and introduced into RAJI cells with shRNA-mediated PHGDH knockdown using retroviral transduction.
